# Contradictory imaging and EEG results in resection surgery of bitemporal lobe epilepsy: A case report

**DOI:** 10.3892/etm.2013.1462

**Published:** 2013-12-27

**Authors:** KANG YANG, JING SU, YUE LANG, SHU-PING LIU, JIAN YIN

**Affiliations:** 1 Department of Neurosurgery, The Second Affiliated Hospital of Dalian Medical University, Dalian, Liaoning 116044, P.R. China; 2 Epileptic Center of Liaoning, The Second Affiliated Hospital of Dalian Medical University, Dalian, Liaoning 116044, P.R. China

**Keywords:** epilepsy, bitemporal lobe epilepsy, epileptic surgery

## Abstract

The present study describes the case of a 27-year-old, right-handed female with bilateral mesial temporal lobe epilepsy. Electroencephalogram (EEG) monitoring from implanted electrodes displayed two different and independent onsets on the two sides of the mesial temporal structures, which specifically included clinical generalized tonic clonic seizure (GTCS) discharges originating from the left mesial temporal lobe, as well as complex partial seizure (CPS) discharges arising from the right mesial region. However, fluid-attenuated inversion recovery magnetic resonance imaging (FLAIR MRI) showed a unilateral abnormality, as in right mesial temporal lobe sclerosis. A decision was made to resect one side of the mesial temporal lobe, in order to avoid memory function impairment, and, relying on the MRI results, the right side was selected. However, surgery did not leave the patient seizure-free. The CPSs gradually eased, while the GTCSs originating from the left side became severely aggravated. In describing this case, the drawbacks of current epileptic diagnostic methods and surgical strategies for bitemporal lobe epilepsy are discussed, and the requirement for more treatment options is emphasized.

## Introduction

Epilepsy is a frequently occurring neurological condition resulting from the sudden discharge of brain neurons. It is the most common serious neurological disorder globally, and adversely affects social, vocational and psychological functioning (1). The prevalence of epilepsy in developed countries ranges from 4 to 10 cases per 1,000 (2). Approximately two-thirds of patients with epilepsy will achieve a seizure-free status with antiepileptic medications; however, one-third of cases are intractable to antiepileptic drugs (AEDs) and therefore require surgical intervention (3). However, surgical treatment fails to provide a seizure-free outcome in 20–30% of patients with temporal lobe epilepsy (TLE) (4). Patients with bitemporal lobe epileptogenic foci are representative of poor candidates for resective surgery due to the difficulty of achieving a favorable outcome (5–7). Independent bitemporal seizure onset is largely assessed using electroencephalography monitoring, which is limited by its duration. The number of seizure recordings required to assure unilateral seizure onset is debatable, and depends on the proportion of seizures from each temporal lobe and the tendency of seizures to cluster from one temporal lobe as a result of a short interseizure interval or other such factors (8–12). In the present study, a rare case of a patient with bitemporal lobe epilepsy, who received unilateral standard temporal lobectomy, is described. In addition, the possible pathogenesis of bitemporal lobe epilepsy is discussed. The present study was approved by the ethics committee of Dalian Medical University (Dalian, China; number 2013-002-08). Informed consent was obtained from all patients.

## Case report

**Figure 1:**
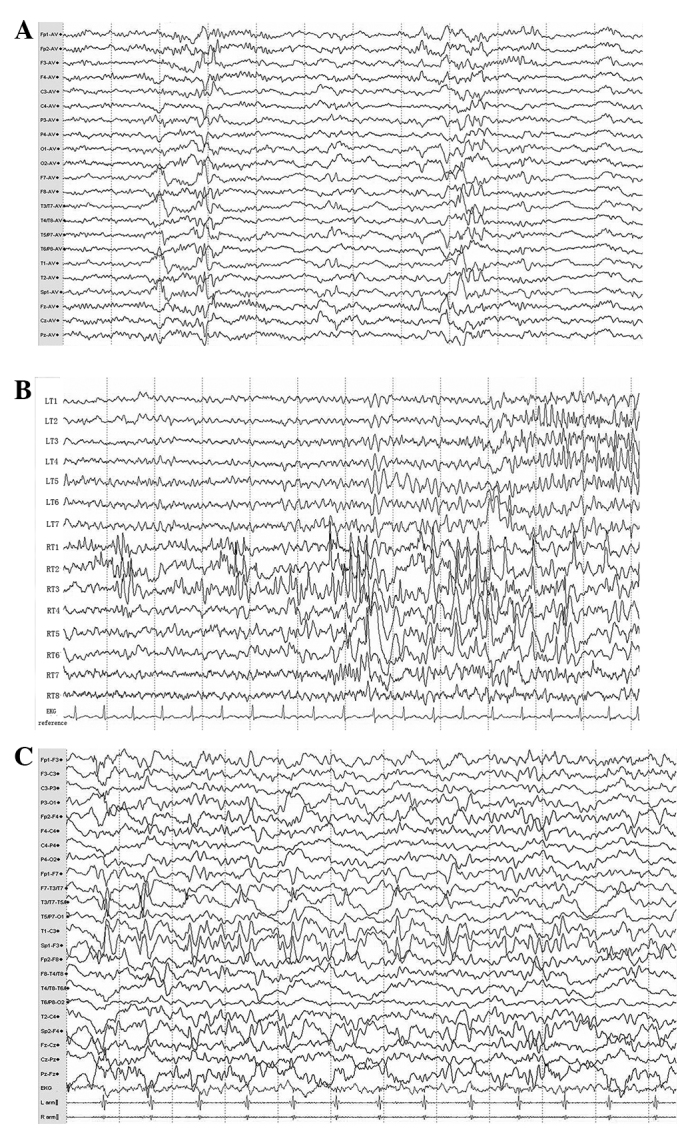
(A) Video-electroencephalogram (V-EEG) monitoring revealed intermittent multiple middle-amplitude spikes rising from the bilateral anterior temporal lobes, particularly in electrodes T1–4 (electrodes T1 and 3 were placed in the left side while T2 and 4 were in the right side). (B) The electrocorticogram (ECoG) monitoring with two depth electrodes in the bilateral temporal lobe revealed two different and independent focal seizure activities arising from the two sides of the temporal lobes as multiple spikes in the left and right temporal electrodes (LT 2–3 and RT 1–3, respectively). (C) Postoperative EEG assessment showed multiple continuous slowing and intermittent epileptiform discharges from the left sphenoid and temporal electrodes (Sp1 and T1, respectively) during the interictal period.

**Figure 2:**
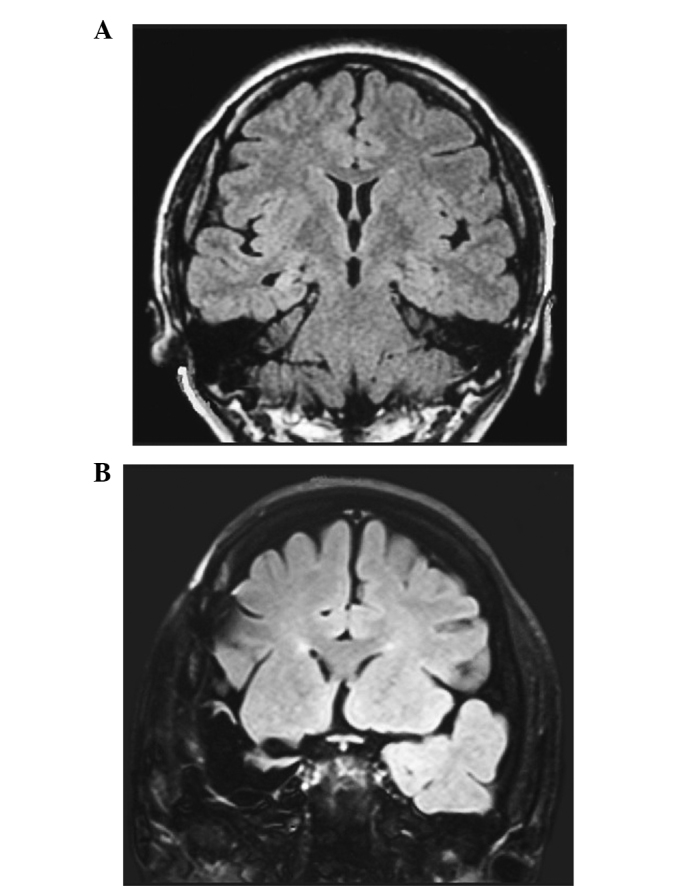
(A) Presurgical fluid-attenuated inversion recovery magnetic resonance imaging (FLAIR MRI) revealed an increased signal in the right mesial temporal structures, suggesting right mesial temporal sclerosis. (B) Postsurgical T1-weighted MRI confirmed the total removal of the right epileptogenic foci.

A 27-year-old, right-handed female who had suffered from recurrent seizures since the age of 11 was referred to the Epileptic Center of Liaoning (The Second Affiliated Hospital of Dalian Medical University) for medically refractory epilepsy. The patient had tried almost all types of AEDs, yet none of them were capable of controlling the seizures. The patient had experienced two types of seizure attacks: One was characterized by a paroxysmal loss of consciousness followed by automatic movements, such as gazing eyes, a sudden halting of action, swallowing or lip smacking, with the symptoms taking ~1 min to ease; the other type also began with an impairment of consciousness, yet evolved into a generalized tonic-clonic seizure (GTCS), which was characterized by the head and eyes deviating to the left side, drooling and limb flexing, shaking and stiffening, which was sustained for 4–5 min and was usually followed by some sleep. Initially, the two types of seizures occurred three to four times; however, this gradually increased to eight to nine times per month. The patient was born a mature infant, but had a history of encephalitis and febrile convulsion at the age of six. Neurological and neuropsychological examinations were normal. Scalp video-electroencephalogram (V-EEG) monitoring revealed intermittent multiple spikes, middle-amplitude waves and interictal spikes from the bilateral anterior temporal lobes (Fig. 1A). Fluid-attenuated inversion recovery magnetic resonance imaging (FLAIR MRI) revealed an increased signal within the right mesial temporal structures, suggesting right mesial temporal sclerosis (Fig. 2A). Fluorodeoxyglucose-positron emission tomography (FDG-PET) imaging showed mild hypometabolism in the bilateral temporal lobes.

Based on the presurgical evaluation, it was suggested that the patient had bitemporal lobe epilepsy. However, the imaging and EEG results did not coincide with regard to the side that required surgical resection. Therefore, cortex and depth electrodes were implanted in the two temporal lobes for surgical evaluation. The five-day electrocorticogram (ECoG) monitoring displayed two different and independent onsets on the two sides of the mesial temporal structures (Fig. 1B), which specifically included 11 typical episodes of GTCSs originating from the left mesial temporal lobe, as well as six clinical episodes of complex partial seizures (CPSs) arising from the right mesial temporal region, which indicated a typical bitemporal lobe epilepsy. Considering the patient’s FLAIR MRI results, a resection of the right anterior temporal lobe, hippocampus and amygdala was performed, and postsurgical MRI confirmed the total removal of the right epileptogenic foci (Fig. 2B). Pathological examination of the surgical specimen confirmed hippocampal sclerosis. However, despite the easing of the patient’s CPSs the GTCSs were aggravated and occurred more frequently. Postsurgical EEG assessment showed epileptiform discharges from the left temporal lobe (Fig. 1C). As a result, the patient currently takes 150 mg lamotrigine and 400 mg phenobarbital daily.

## Discussion

This case is notable due to the contradiction between the imaging and EEG results. A previous study by Van Ness *et al* (13) estimated that 17 serial seizures were required to be recorded to confidently establish that <20% of seizures arose from a second site (13). Subsequently, Blum (8) used Bayes’ theorem and selected data from an epilepsy monitoring unit to constrain the statistical possibilities and estimate that recording five serial seizures from a single focus confidently established that site as the sole focus (8). In the present case, scalp EEG displayed equal discharges from the bilateral temporal lobe and the implanted electrodes further confirmed the diagnosis. However, FLAIR MRI showed a unilateral abnormality. This contradiction enhanced the difficulty of selecting the optimal surgical strategy for this case. Despite this, it was decided to resect one side of the mesial temporal lobe only, in order to avoid memory function impairment. Relying on the MRI results, the right side was selected. However, surgery did not leave the patient seizure-free. Even though the CPSs arising from the right side gradually eased, the GTCSs originating from the left side became severely aggravated.

In normal circumstances, noninvasive methods are used to provide a diagnosis of bitemporal lobe epilepsy and invasive intracranial electrodes are able to suggest the predominance of unitemporal seizure onset (14). However, traditional invasive monitoring methods are limited by the duration of the recordings (7). Patients with bitemporal lobe epilepsy may cycle seizures from one side to the other side at various intervals (5), and the proportion of seizures arising from one temporal lobe during the standard invasive evaluation is not a reliable prognostic factor of epilepsy surgery (15). Therefore, the selection of the resection side during a limited period of the recording may be challenging. It is not possible to overcome this problem of sampling with short-term monitoring, and a long-term invasive evaluation is likely to be beneficial in these patients (16–18). This may be one primary reason that the correct side for resection was not selected in the present case.

In describing this case, it is suggested that clinicians should pay more attention to the patients’ symptoms when confronted with similar cases, as GTCSs have a greater effect on the patients’ quality of life. Furthermore, clinicians should consider alternative options, such as responsive cortical stimulation. This is a recently developed method for the treatment of epilepsy that reduces the frequency of disabling partial seizures and monitors epileptic discharges long enough to judge the side of the majority of the seizures, therefore enabling the modification of the surgical strategy (5,16–18,19). If epileptic activities of either side arise from the temporal neocortex, it is suggested that bipolar coagulation on functional cortices (BCFC) or multiple subpial transection (MST) are combined with selective amygdalohippocampectomy (SAH), respectively, according to the sites of the epileptogenic foci, i.e., in the amygdalohippocampal complex or temporal neocortex (20,21). Furthermore, it is suggested that additional points of view are sought in the treatment of similar cases of epilepsy.
